# IMPACT OF VITAMIN D AND CALCIUM DEFICIENCY IN THE BONES OF PATIENTS UNDERGOING BARIATRIC SURGERY: A SYSTEMATIC REVIEW

**DOI:** 10.1590/0102-6720201600S10029

**Published:** 2016

**Authors:** Jefry Alberto Vargas CABRAL, Gabriela Pereira de SOUZA, Juliana de Almeida NASCIMENTO, Luis Fernando SIMONETI, Carolina MARCHESE, Silvia Helena de Carvalho SALES-PERES

**Affiliations:** 1 Postgraduate Program in Public Health Dentistry; University of São Paulo, SP, Brazil.; 2 Postgraduate Program in Biological Sciences; University of São Paulo, SP, Brazil.; 3 Department of Pediatric Dentistry, Orthodontics and Public Health, Bauru School of Dentistry, University of São Paulo, SP, Brazil.

**Keywords:** Bariatric surgery, Obesity, Absorption, Bone, Vitamin D, Calcium

## Abstract

**Introduction:**

Bariatric surgery is considered the most effective tool in the control and treatment of severe obesity, but patients undergoing this procedure are at increased risk of developing nutritional deficiencies by limiting the intake and absorption of many nutrients.

**Objective:**

To assess the impact of vitamin D deficiency and calcium in bone in patients after gastric bypass in Roux-en-Y, pointing directly at the type of administration, doses and effects after surgery.

**Method:**

Was conducted a systematic review with articles related to the topic of the last 10 years searched in PubMed (US National Library of Medicine National Institutes of Health, Medline, Lilacs, Scielo and Cochrane using the headings "bariatric surgery", "bone", "obesity", "vitamin D '', "calcium" AND "absorption". Exclusion criteria to research on animals, smokers, pregnant women and patient treated with bisphosphonates.

**Results:**

Five articles were included in this review. All refer that bariatric surgery can lead to nutritional deficiencies and poor absorption of fats and fat-soluble vitamins and other micronutrients such as calcium.

**Conclusion:**

Patients submitted to RYGB should make use of multivitamins and minerals especially vitamin D and calcium to prevent bone fractures. Monitoring, treatment and control of risk factors are essential to prevent complications after this operation.

## RESUMO

**Introdução:**

A cirurgia bariátrica é considerada o tratamento mais eficaz no controle e tratamento da obesidade severa; porém, indivíduos submetidos a este procedimento apresentam maior risco de desenvolver deficiências nutricionais pela limitação na ingestão e absorção de muitos nutrientes.

**Objetivo:**

Avaliar o impacto da deficiência de vitamina D e do cálcio ósseo de pacientes após gastroplastia em Y-de-Roux, relacionando tipo de administração, dose e efeito pós-cirúrgico.

**Método:**

Realizou-se revisão sistemática com artigos relacionados ao tema dos últimos 10 anos e pesquisados na PubMed (US National Library of Medicine National Institutes of Health, Medline, Lilacs, Scielo e Cochrane usando os descritores "bariatric surgery", "bone", "obesity", " vitamin D'', "calcium'' AND "absortion". Foram excluídos os estudos em animais, fumantes, grávidas ou gestantes e indivíduos que ingeriram bisfosfonatos.

**Resultados:**

Foram incluídos ao final cinco artigos. Todos referem que a cirurgia bariátrica pode levar à deficiências nutricionais como má absorção de gorduras, vitaminas lipossolúveis, micronutrientes e cálcio.

**Conclusão:**

Os pacientes submetidos ao RYGB devem fazer uso de polivitamínicos e minerais principalmente a vitamina D e o cálcio para evitar fraturas ósseas. Monitoramento, tratamento e controle dos fatores de risco são essenciais para prevenir estas complicações após a operação.

## INTRODUCTION

Since the development of bariatric surgery, many surgical methods for the treatment of morbid obesity have been developed over the past decades. The Roux-Y gastric bypass (RYGB) is an operation which is considered gold standard treatment alternative for severe obesity^3^ because it promotes less severe absorption and complications than traditional malabsorption procedures, such as jejunoileal bypass^3^ ^,^ ^15^ ^,^ ^26^. The malabsorption procedures have been recognized as a risk factor for the development of bone^5^ ^,^ ^8^ ^,^ ^12^ ^,^ ^17^ ^,^ ^28^ disease as a result of modification of calcium (Ca) metabolism and impairment of its absorption^4^ ^,^ ^7^ ^,^ ^14^ ^,^ ^16^ ^,^ ^18^ ^,^ ^22^ ^,^ ^23^. Only a few studies have investigated the absorption of Ca prospectively in patients with jejunoileal bypass and showed that absorption decreases by 50% after surgery^7^ ^,^ ^14^ ^,^ ^22^. To our knowledge, the change in Ca absorption after RYGB surgery has not been addressed previously. In addition, inadequate intake Ca is common after gastric bypass^1^ ^,^ ^20^, which can also contribute to altered bone loss^6^.

Understand the postoperative RYGB decrease in absorption and calcium intake and investigate the doses, routes of administration, the time of drug therapy and its effects on bone, were the objectives of this review.

## METHODS

Was adopted the PICO method (population, intervention, comparison and outcome) to elaborate the answer for this question "How is bone loss in patients who undergo bariatric surgery and what supplements help to decrease this loss?"

Were enrolled obese patients who had bone loss and or BMI from 35-39.9 kg/m^2^ with comorbidities and ≥40 kg/m^2^ (population); patients submitted to RYGB by laparoscopy or laparotomy (intervention); eutrophic patients with 18.5-24.9 kg/m^2^ (comparison); deficiency of vitamin D and calcium, possible presence of fractures (outcome).

### Eligibility criteria for study inclusion

Inclusion criteria were: all studies; patients from 15-70 years; BMI from 35-39.9 kg/m^2^ with comorbidities and ≥40 kg/m^2^; postoperative at least of three months; laparoscopic or laparotomic RYGB. Exclusion criteria were: pregnant women or women in stage of lactation; smoking or former smoker; individuals treated with bisphosphonates; studies in animals.

### Types of outcome

Primary result was focused in vitamin D and calcium deficiency; however, was explored possible bone fractures after bariatric surgery. As secondary results, was searched relationship between the type of administration and the body's efficiency in absorbing medication; and the doses and its effects on the maintenance or recovery of bone loss after bariatric surgery.

### Search strategy

PubMed/Medline, Lilacs, Scielo and Cochrane were used crossing the headings "bariatric surgery", "bone", "obesity", "vitamin D '', "calcium" "AND" "absorption". After the search, analysis of the title, reading the abstract and finally the complete reading of the articles has been made.

### Screening methods

Four reviewers made the primary research for titles and abstracts. Afterwards, the same reviewers assessed the full manuscript observing compliance with the inclusion/exclusion criteria or those with insufficient data in the title and abstract. Any disagreement was resolved by discussion between the reviewers and an independent reviewer conducted a manual search.

When the results of a study were published more than once and the results were presented in various publications by the same author, they were included only once in this review.

## RESULTS

**FIGURE 1:**
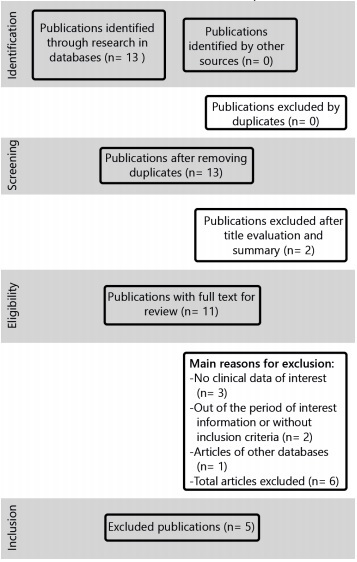
Flowchart describing the strategy of search and selection process

Flowchart (Figure 1) illustrates the strategy of search and selection process of the 13 titles identified by electronic search. Six were discarded by titles and abstracts, resulting in seven studies, which underwent full-text analysis. Afterwards, two publications were excluded for not meeting the inclusion criteria. At the end, remained five articles that were analyzed for this review. 

### Study description

Table 1 shows the methodological characteristics of selected studies. Of the five articles, three corresponded to prospective cohort studies; two used laparoscopic approach and one enrolled only women; there was one case report; one case series. All assessed the nutritional status and bone fractures, routes of administration, as well as the respective dosage of vitamin D and calcium; one article evaluated the parathyroid hormone and its influence on bone reabsorption in RYGB.

**TABLE 1:** Methodological characteristics of the selected studies, type of interventions and results

Reference (year)	Type of study	Study location	Period of follow-up	N *	Tipe of surgery **	age	Sex***	Type of medication	Type of administration	Individual	Doses	Time of medical therapy	Conclusion
Vasconcelos RS et. al 2010 ^25^	Case Series	Brazil	From 7 to 22 months	n=29	RYGB	>18 years	F	Calcium e Vitamin D	(tablets and diet)	people	600mg Diet +200mg tablet of Ca and 500 IU Vitamin D (per day)	From 7 to 22 months	There were no significant differences between the average bone mass density and prevalence of vertebral fractures in both groups.
Flores L. et. al 2010 ^10^	Prospective cohort study	Espain	1 year	n=222	RYGBL	18-65 Years	F e M	Calcium and Vitamin D	(tablets)	people	1,200 mg Ca and 800 IU Vitamin D (per day)	4,8 e 12 months	The parathyroid hormone leads cortical bone destruction and improving serum Ca. 80% of patients have vitamin D deficiency but not bone fractures.
Williams SE. et al 2008 ^27^	Case study	United States	2 years	N=1	RYGB	56 years	F	Cálcium vitamin D	(tablets)	person	500mg Ca and 400 IU (per day)	2 years	After 2 years the patient showed no fracture or risk to bone fracture level.
Avgerinos DV. et al 2007 ^2^	A prospective cohort study	United States	2 years	N=444 (M=91 F=353)	RYGB	21-64 years	M e F	Cálciume vitamin D	(tablets)	people	1,200 mg Ca and 800IU VitaminaD	1.8 years	Total calcium decreases in body related mobilization of bone. Supplementation with vitamin D prevent the decrease in bone calcium.
Riedt CS et. al 2006 ^19^	A prospective cohort study	Unites States	6 months	N=21	RYGB and RYGBL (5:open field and 16 laparoscopy)	29-62 years	F	Cálcium Vitamin D	(tablets, diet and injected)	people	Diet, 1,000 mg of Ca and 400 IU Vitamin D	6 months	Low Ca absorption after surgery, being considered markers of bone resorption (60 to 200%). There was a higher bone resorption than bone formation

## DISCUSSION

The results of this systematic review are based in five publications. The research did not identify bone fractures in patients undergoing bariatric surgery; however, showed high deficiencies in vitamin D and calcium in the bones. The studies showed the different routes of administration, and the results, answering the various questions about deficiencies that arise in the bone tissue due to dosage and effectiveness, according to the route of administration.

After bariatric surgery all investigations reported results with deficiency in the bones of patients, regardless of the type of bariatric surgery (videolaparoscopy or laparotomy). All mentioned intake of vitamin D and calcium in the diet, via tablets or injections in different dosage.

 None of them presented complete elimination of bone loss, but showed a significant difference in bone resorption, mainly by parathyroid hormone, which increases the activity of osteoclasts leading to the destruction of the cortical bone, showing also marked deficiency of vitamin D^10^ ^,^ ^23^, weakening the bones and the possibility of fractures in postoperative period. Avgerinos et al., 2007 ^2^ in his important prospective cohort in individuals of both genders for two years have shown the importance of vitamin D supplementation to prevent the decrease of calcium in the bones. Other authors also showed high deficiency of vitamin D in patients after this surgery ^2^ ^,^ ^9^ ^,^ ^10^ ^,^ ^19^ ^,^ ^25^ ^,^ ^27^.

Researchers analyzed women in pre- and postmenopausal stage showing that there was no significant difference in calcium absorption deficiency and even differences in relationship to the type of surgery^20^ ^,^ ^25^ ^,29^.

According to this review the types of administration and dosage had no relationship or relevance over time on drug therapy. However, showed no direct relation to the postoperative bone loss. Vasconcelos et al. consider the calcium intake in the diet at 600 mg and supplemented with 200 mg in tablets during the 22 months in the operated group. Although significantly higher than in the non-operated group was still lower than recommended levels for these patients, that should be between 1000-1800 mg/day^11^ ^,^ ^13^ ^,^ ^24^. Intake of vitamin D (500 IU) was also below the recommended levels.

It can be inferred from that the postoperative vitamin supplementation should not only consist of multivitamins, because most do not contain the calcium and vitamin D required and recommended to be taken every day. The above changes may increase during postoperative and preoperative screening; care should be taken to prevent the changes in bone metabolism. Suitable supplementation of vitamins and minerals is essential to prevent or minimize bone metabolic complications that can occur after RYGB^25^.

An important factor in addition to vitamin D supplementation and calcium that may affect bone change in these patients is the age, besides the differences between women in premenopausal and postmenopausal women that need specific approach.

There are other factors that can influence directly and contribute to bone resorption, which is a chronic deficiency of vitamin D, inadequate calcium intake and secondary hyperparathyroidism appearing sometimes in obese. The parathyroid hormone also increases the activity of osteoclasts leading to bone cortical destruction to compensate for the decrease of serum Ca^10^.

In relation to bone density and fracture prevalence no significant differences in the studies were found. It is possible that the relatively short follow-up contributed to the lack of identification of bone fracture. Future long-term studies should be conducted to better clarify the bone complications in these patients.

## CONCLUSIONS

Patients undergoing RYGB should make use of multivitamins and minerals especially calcium and vitamin D to prevent bone fractures. Monitoring, treatment and control of risk factors are essential to prevent these complications after the surgery.
